# Dissection of additive, dominance, and imprinting effects for production and reproduction traits in Holstein cattle

**DOI:** 10.1186/s12864-017-3821-4

**Published:** 2017-05-30

**Authors:** Jicai Jiang, Botong Shen, Jeffrey R. O’Connell, Paul M. VanRaden, John B. Cole, Li Ma

**Affiliations:** 1 Department of Animal and Avian Sciences, University of Maryland, 2123 Animal Science Building, College Park, MD 20742 USA; 2 University of Maryland Baltimore, Baltimore, MD 21201 USA; 3 Animal Genomics and Improvement Laboratory, USDA, Building 5, Beltsville, MD 20705 USA

**Keywords:** Variance decomposition, Additive, Dominance, Imprinting, Cattle, Dairy traits, Qtl

## Abstract

**Background:**

Although genome-wide association and genomic selection studies have primarily focused on additive effects, dominance and imprinting effects play an important role in mammalian biology and development. The degree to which these non-additive genetic effects contribute to phenotypic variation and whether QTL acting in a non-additive manner can be detected in genetic association studies remain controversial.

**Results:**

To empirically answer these questions, we analyzed a large cattle dataset that consisted of 42,701 genotyped Holstein cows with genotyped parents and phenotypic records for eight production and reproduction traits. SNP genotypes were phased in pedigree to determine the parent-of-origin of alleles, and a three-component GREML was applied to obtain variance decomposition for additive, dominance, and imprinting effects. The results showed a significant non-zero contribution from dominance to production traits but not to reproduction traits. Imprinting effects significantly contributed to both production and reproduction traits. Interestingly, imprinting effects contributed more to reproduction traits than to production traits. Using GWAS and imputation-based fine-mapping analyses, we identified and validated a dominance association signal with milk yield near *RUNX2*, a candidate gene that has been associated with milk production in mice. When adding non-additive effects into the prediction models, however, we observed little or no increase in prediction accuracy for the eight traits analyzed.

**Conclusions:**

Collectively, our results suggested that non-additive effects contributed a non-negligible amount (more for reproduction traits) to the total genetic variance of complex traits in cattle, and detection of QTLs with non-additive effect is possible in GWAS using a large dataset.

**Electronic supplementary material:**

The online version of this article (doi:10.1186/s12864-017-3821-4) contains supplementary material, which is available to authorized users.

## Background

Both dominance and imprinting play an important role in mammalian biology and development [1]. Though one may naturally assume that dominance and imprinting effects affect economically important traits in plants and animals, it remains controversial how much phenotypic variation can be attributed to these non-additive effects, how many quantitative trait loci (QTL) follow non-additive inheritance, and whether incorporating non-additive genetic effects will benefit genomic prediction [2–4]. Generally, contribution of non-additive genetic effects varies for different types of traits. For example, genetic variation associated with fitness-related traits is due mostly to low frequency, deleterious variants, so these traits typically show relatively high non-additive variance out of the total genetic variation [2].

Several studies have been conducted to decompose dominance genetic effects from the total genetic variance of complex traits, theoretically [5–8] and empirically [9–12]. A few recent studies have tried to add imprinting effects into the decomposition of total genetic variation [13–16]. These studies indicated that non-additive effects have a significant contribution to the total genetic variance, but it is still questionable whether or not this contribution can be robustly translated into more accurate genomic prediction in real populations. More recently, it was shown that mating programs increased rates of genetic gain when non-additive genetic effects were included [17–19]. Further understanding of the contribution of non-additive effects to the genomic prediction and mating allocation programs will benefit livestock production in the long term.

Gene mapping studies have primarily focused on genetic variants with additive effects. Although many empirical studies have reported non-negligible contributions from non-additive effects to complex traits, QTLs with non-additive effects are still difficult to identify in animal and human gene mapping studies, largely due to the low statistical power in the testing for non-additive effects of individual loci [20]. The large dairy genomics database maintained by the Council on Dairy Cattle Breeding (CDCB) and the USDA Animal Genomics and Improvement Laboratory (AGIL; Beltsville, MD) represents a powerful dataset for mapping QTLs with non-additive effects.

To empirically address questions related to dominance and imprinting effects of complex traits, we analyzed a large cattle dataset that consisted of more than 40 K Holstein cows with SNP genotypes, pedigree information, and eight yield deviation (YD) phenotypes (milk yield, fat yield, protein yield, daughter pregnancy rate, cow conception rate, heifer conception rate, somatic cell score, and productive life). Both parents of these cows were also genotyped to phase the parental inheritance of SNPs of the cows. The aims of this study were to estimate the relative contribution of additive, dominance, and imprinting effects to dairy production and reproduction traits, to identify QTLs with dominance or imprinting effects, and to investigate whether adding these non-additive genetic components improves the prediction accuracy of genomic selection in real data.

## Results

### Variance decomposition of additive, dominance, and imprinting effects

Using 42,701 Holstein cows with YD phenotypes, SNP genotypes, and two genotyped parents, we decomposed the total genetic value of eight dairy traits into additive, dominance, and imprinting effects, estimating corresponding variance components (Table 1). For the eight traits analyzed, the number of animals with YD phenotype ranged from 12,911 (productive life) to 29,811 (milk, fat, and protein yields). Overall, production traits (milk, fat, and protein yields) exhibited a different pattern from reproduction traits (daughter pregnancy, cow conception, and heifer conception rates). As shown in Table 1, the broad-sense heritability (*H* ^2^ = proportion of total genetic variance in phenotypic variance) was 31.9–38.6% for production traits and 1.4–7.9% for reproduction traits, respectively. The narrow-sense heritability (*h* ^2^ = proportion of additive genetic variance in phenotypic variance) was 27.2–33.8% for production traits and only 0.8–5.1% for reproduction traits, respectively. Proportions of dominance variance in phenotypic variance were significantly higher (*P* < 0.05) for production traits (2.5%–4.0%) than for reproduction traits (0.2%–1.1%), but the proportions in total genetic variance are higher for reproduction traits. The variance explained by imprinting effect was very low for all eight traits, <1% of the phenotypic variance for production traits and 1–2% for reproduction traits. However, these imprinting effects were significantly larger than zero for most production and reproduction traits (*P* < 0.05). Moreover, for reproduction traits that have a low heritability, imprinting effects explained a relatively large portion of the total genetic variance (20.9% for daughter pregnancy rate, 26.4% for cow conception rate, and 35.4% for heifer conception rate), which were significantly higher than those for production traits (*P* < 0.05).

**Table 1:** Variance decomposition of genotypic additive, dominance, and imprinting values for eight dairy traits

Trait	*N*	Proportion in phenotypic variance (SE)				Proportion in total genetic variance			*P*-value of test for σ^2^ = 0		
		A	D	I	*H* ^2^	A	D	I	A	D	I
MY	29,811	0.338 (0.009)	0.040 (0.005)	0.008 (0.002)	0.386 (0.009)	0.875	0.104	0.020	3.5 × 10^−151^	9.9 × 10^−15^	4.9 × 10^−4^
FY	29,811	0.312 (0.009)	0.025 (0.005)	0.004 (0.002)	0.340 (0.009)	0.917	0.073	0.010	3.9 × 10^−145^	1.1 × 10^−7^	0.04
PY	29,811	0.272 (0.009)	0.040 (0.005)	0.007 (0.002)	0.319 (0.009)	0.853	0.126	0.021	1.8 × 10^−122^	1.3 × 10^−13^	2.5 × 10^−3^
SCS	29,392	0.102 (0.007)	0.010 (0.006)	0.002 (0.002)	0.114 (0.007)	0.893	0.087	0.019	2.2 × 10^−48^	0.04	0.14
STPL	12,911	0.031 (0.007)	0.000 (0.011)	0.000 (0.004)	0.031 (0.010)	1.0	0.0	0.0	3.4 × 10^−06^	0.5	0.5
DPR	22,942	0.044 (0.006)	0.011 (0.007)	0.015 (0.004)	0.069 (0.008)	0.637	0.154	0.209	5.2 × 10^−15^	0.07	1.9 × 10^−5^
CCR	14,318	0.051 (0.008)	0.007 (0.011)	0.021 (0.005)	0.079 (0.011)	0.647	0.090	0.264	2.2 × 10^−11^	0.27	6.0 × 10^−5^
HCR	28,601	0.008 (0.003)	0.002 (0.005)	0.005 (0.002)	0.014 (0.005)	0.538	0.108	0.354	3.5 × 10^−3^	0.39	0.01

*MY* milk yield, *FY* fat yield, *PY* protein yield, *SCS* somatic cell score, *STPL* standardized productive life, *DPR* daughter pregnancy rate, *CCR* cow conception rate, *HCR* heifer conception rate, *N* sample size, *A* additive effect, *D* dominance effect, *I* imprinting effect, *SE* standard error, *H* ^2^ broad-sense heritability

**Fig. 1:**
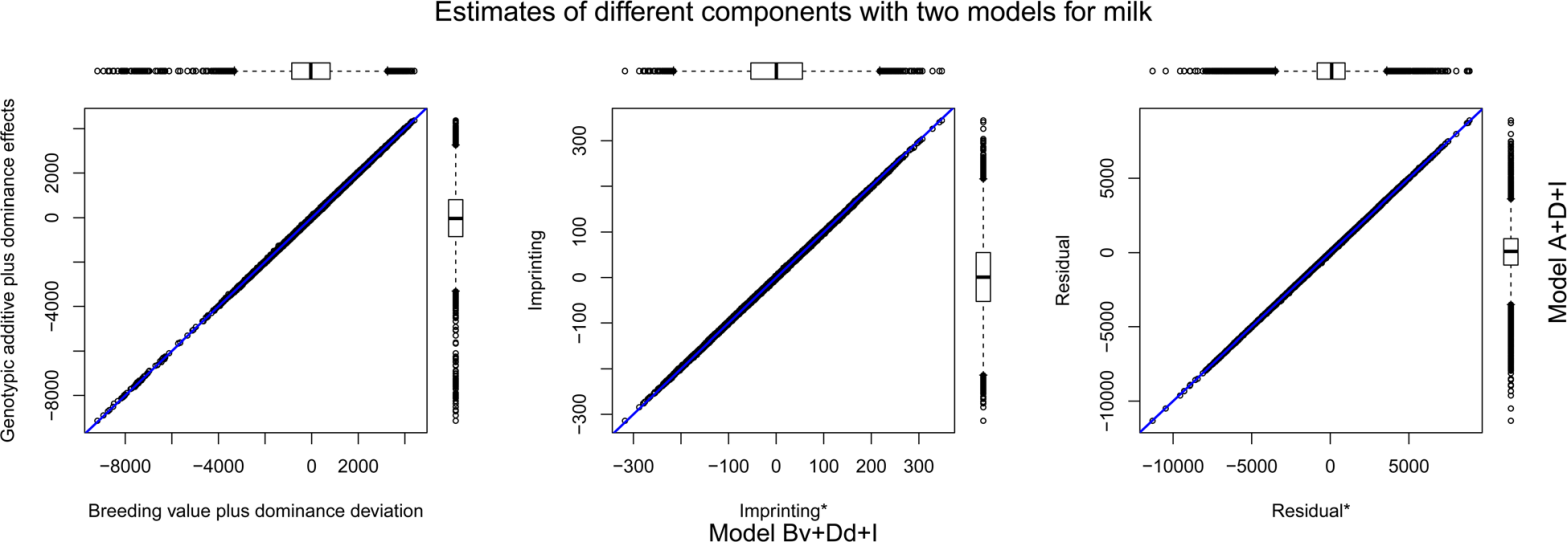
Individual estimates of variance components with two decomposition models for milk. Each point indicates the component estimate for each individual. Blue line indicates *y = x*. The x-axis shows the components from the model decomposing genetic effect to breeding value, dominance deviation and genotypic imprinting value, while y-axis shows the components from the model decomposing genetic effect to genotypic additive, dominance and imprinting values

For comparison purposes, the total genetic variance was decomposed into the genotypic imprinting value plus either breeding value and dominance deviation using a classical model that considered allele frequencies [6] or additive and dominance effects that did not consider allele frequencies (see Methods). As shown in Additional file 1: Table S1, results from these two decomposition models were consistent. It is worth noting that estimated *H* ^2^ from the two models was exactly the same for all eight traits. In addition, the proportion of variance explained by imprinting effects was the same for the two models. These results were consistent with theoretical expectations [6, 21]. In theory, the two variance decomposition models are equivalent to each other with the same predicted phenotypic values and residuals. First, the sum of additive and dominance genetic variances is equal to the sum of the variances of breeding value and dominance deviation, under a few common assumptions (see Methods). With a stronger condition, the sum of individual breeding value and dominance deviation will be equal to the sum of individual genotypic additive and dominance values. Second, individual genotypic imprinting values of the two models are the same, asserting an equivalence of imprinting variance components. We observed all of these results across all eight traits, as shown in Fig. 1 for milk and Additional file 2: Figure S1 for other traits. Additionally, we confirmed that individual residual estimates of the two models are the same (see the right panels in Fig. 1 and Additional file 2: Figure S1).

**Fig. 2:**
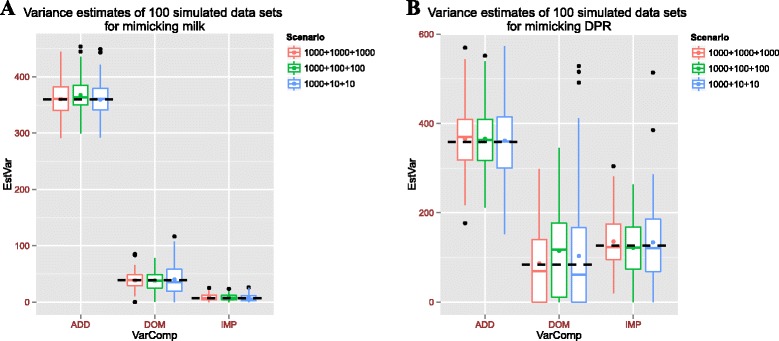
Variance decomposition using simulated datasets. The dash line indicates expected value of corresponding variance component. **a** Variance estimates of 100 simulated data sets for mimicking milk. **b** Variance estimates of 100 simulated data sets for mimicking DPR

Genomic relationship matrix (GRM) based variance decomposition is highly dependent on the assumption of polygenic genetic architecture, as genome-wide SNP genotypes are used with equal weights. Existing GWAS have provided evidence of a polygenic architecture of additive effects in most complex traits [22]. However, we have no such knowledge for dominance and imprinting effects. To investigate the influence of this polygenic assumption on variance components estimation, we performed simulations to determine if our models have biases when there are only a few dominance or imprinting QTLs. Simulation results showed that GREML could accurately estimate variances for genotypic dominance and imprinting values for a moderate-heritability trait like milk yield, even when only 10 dominance and imprinting QTLs were simulated for a trait with polygenic additive effects, respectively (Fig. 2a). For a low-heritability trait like daughter pregnancy rate, GREML also performed well for both lowly and highly polygenic architectures of dominance and imprinting effects (Fig. 2b). Using simulation, we demonstrated the robustness of our approach to the assumption of polygenic genetic architecture.

### Genome-wide association study of dominance and imprinting effects

**Fig. 3:**
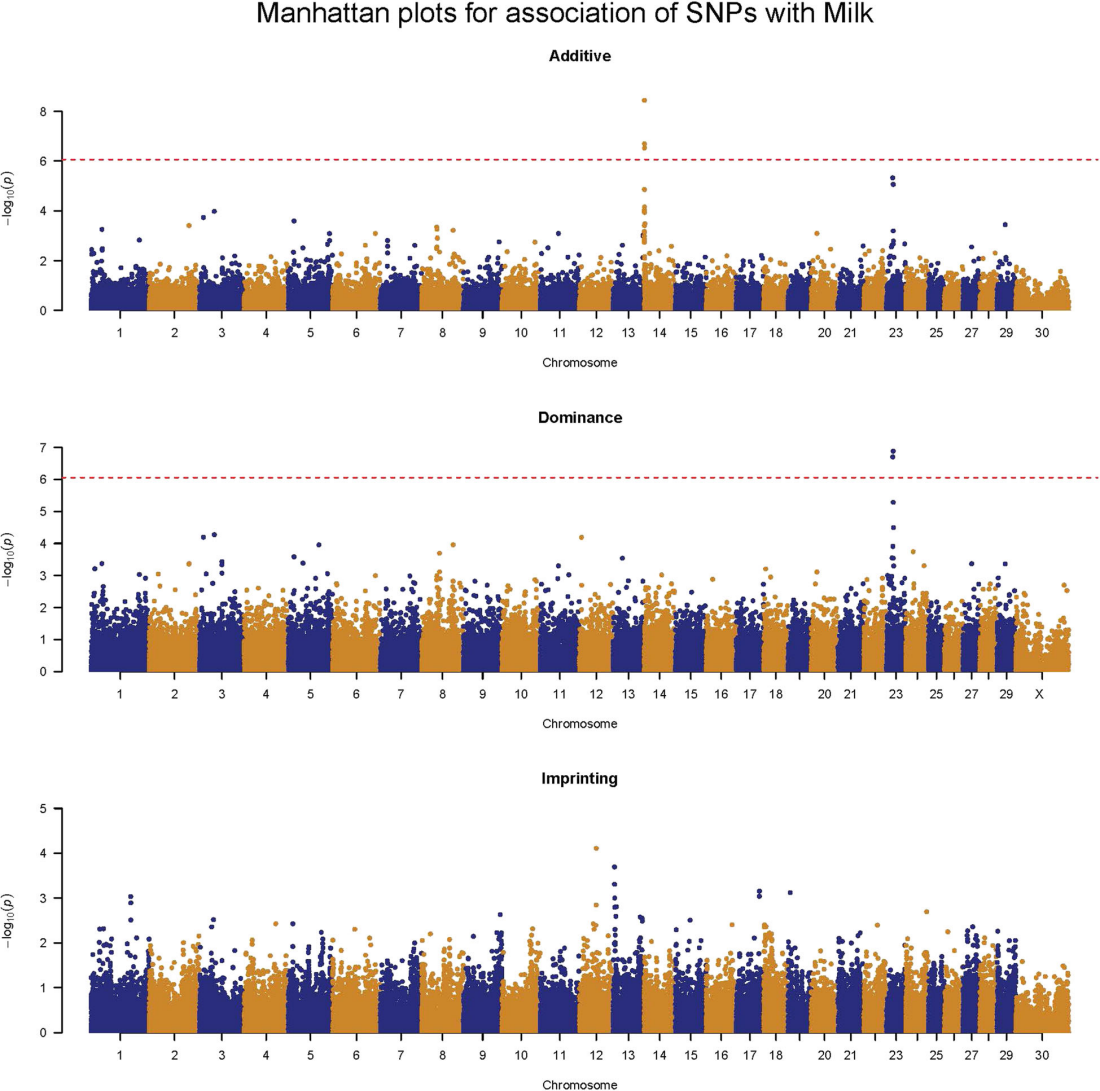
Manhattan plots for associations of SNP effects with milk yield

We performed a whole-genome single-marker scan for additive, dominance, and imprinting effects on all eight traits. To increase computational efficiency, we used a two-step approach to remove polygenic effects from the data: 1) a mixed model with genomic relationship matrices to generate residuals; followed by, 2) a GWAS scan using residuals from the mixed model as the phenotype. Although our two-step strategy has slightly lower power than a single-step mixed model, we identified a novel dominance signal on chromosome 23 that was associated with milk yield (Fig. 3). We then used a single-step mixed model to re-analyze the SNPs near the dominance signal, generating appropriate results for the associated SNPs (Table 2). The top 2 SNPs, Hapmap48809-BTA-55698 and BovineHD2300004730, showed a strong dominance association with milk yield with *P* = 9.54 × 10^−8^ and *P* = 6.33 × 10^−8^, respectively. BovineHD2300004730 is 71 kb upstream of the *RUNX2* gene. The *RUNX2* gene has been previously reported to be a novel regulator of mammary epithelial cell fate in development and breast cancer, and it has also been shown that exogenous transgenic expression of *RUNX2* in mammary epithelial cells blocked milk production [23].

**Table 2:** Top two SNPs associated with milk yield near the *RUNX2* gene

SNP	Chr	Position	MAF	Model	β_A (SE)	*P*-value	β_D (SE)	*P*-value	β_I (SE)	*P*-value
Hapmap48809-BTA-55698	23	17,275,448	0.15	Two-step	153.4 (33.0)	3.33 × 10^−6^	197.1 (37.6)	1.56 × 10^−7^	−3.64 (18.0)	0.84
				A	223.6 (51.8)	1.57 × 10^−5^	255.2 (44.7)	1.17 × 10^−8^	−1.54 (23.8)	0.95
				A + D + I	212.7 (51.6)	3.82 × 10^−5^	241.7 (45.3)	9.54 × 10^−8^	−0.52 (25.5)	0.98
BovineHD2300004730	23	18,600,456	0.10	Two-step	207.3 (47.6)	1.31 × 10^−5^	273.5 (52.1)	1.54 × 10^−7^	10.31 (21.3)	0.63
				A	206.2 (67.6)	2.29 × 10^−3^	353.6 (62.3)	1.43 × 10^−8^	−3.43 (28.8)	0.91
				A + D + I	200.6 (67.5)	2.96 × 10^−3^	340.4 (62.9)	6.33 × 10^−8^	7.52 (30.8)	0.81

*Chr* chromosome, *MAF* minor allele frequency, *β* regression coefficient, *SE* standard error

**Fig. 4:**
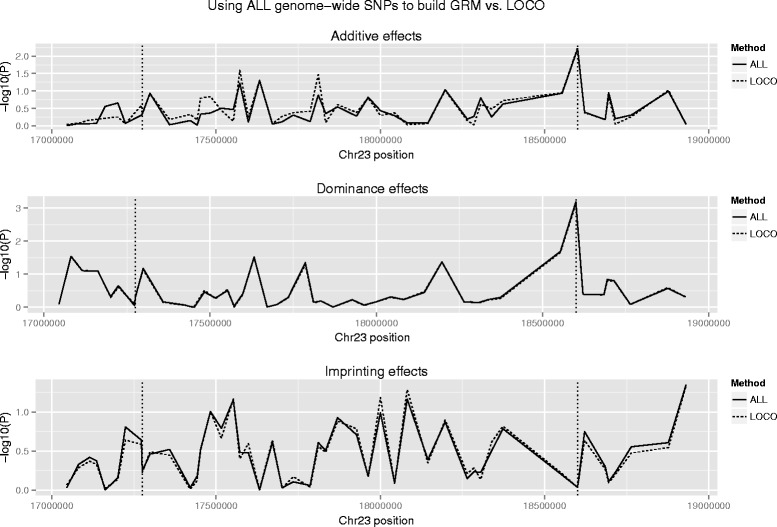
Mixed-model based association analysis between milk yield and 50 SNPs around *RUNX2* in the validation data set. The two vertical dash lines indicate SNPs Hapmap48809-BTA-55698 and BovineHD2300004730, respectively

We further used an independent validation data set consisting of ~5500 younger cows with both genotypes and milk yield phenotypes, which were collected after the initial analysis, to validate the dominance signal associated with milk yield. A mixed-model based method was used to test the association between milk yield and 50 SNPs around the peak signal. This validation analysis provided clear statistical evidence for the dominance association at BovineHD2300004730 with milk yield (*P* = 7.41 × 10^−4^; Fig. 4 and Additional file 3: Table S2). Additionally, we found that the dominance effect was slightly larger than the additive effect at BovineHD2300004730 in both the discovery and validation data sets, suggesting complete dominance or even over-dominance inheritance of the underlying QTL.

We found no other significant non-additive effects for any trait using a genome-wide significance level of 1 × 10^−6^ (Additional file 4: Figure S2). Nevertheless, there were a few nominally significant peaks for dominance or imprinting effects shown in the Manhattan plots, such as the peak for imprinting effect on chromosome 6 for somatic cell score (Additional file 2: Figure S2C) and the one at the end of chromosome 10 for cow conception rate (Additional file 2: Figure S2F). Since a one-step mixed model is more powerful than a two-step scan, we selected 10 nominally significant non-additive association signals and used a one-step mixed-model to test the associations for the top three SNPs within each peak. This one-step re-analysis found a genome-wide significant dominance association on chromosome 10 with both fat and protein yields (Additional file 5: Table S3). However, this dominance signal was not confirmed in the validation data set (Additional file 6: Table S4).

### Fine-mapping of the dominance GWAS peak near *RUNX2*

**Fig. 5:**
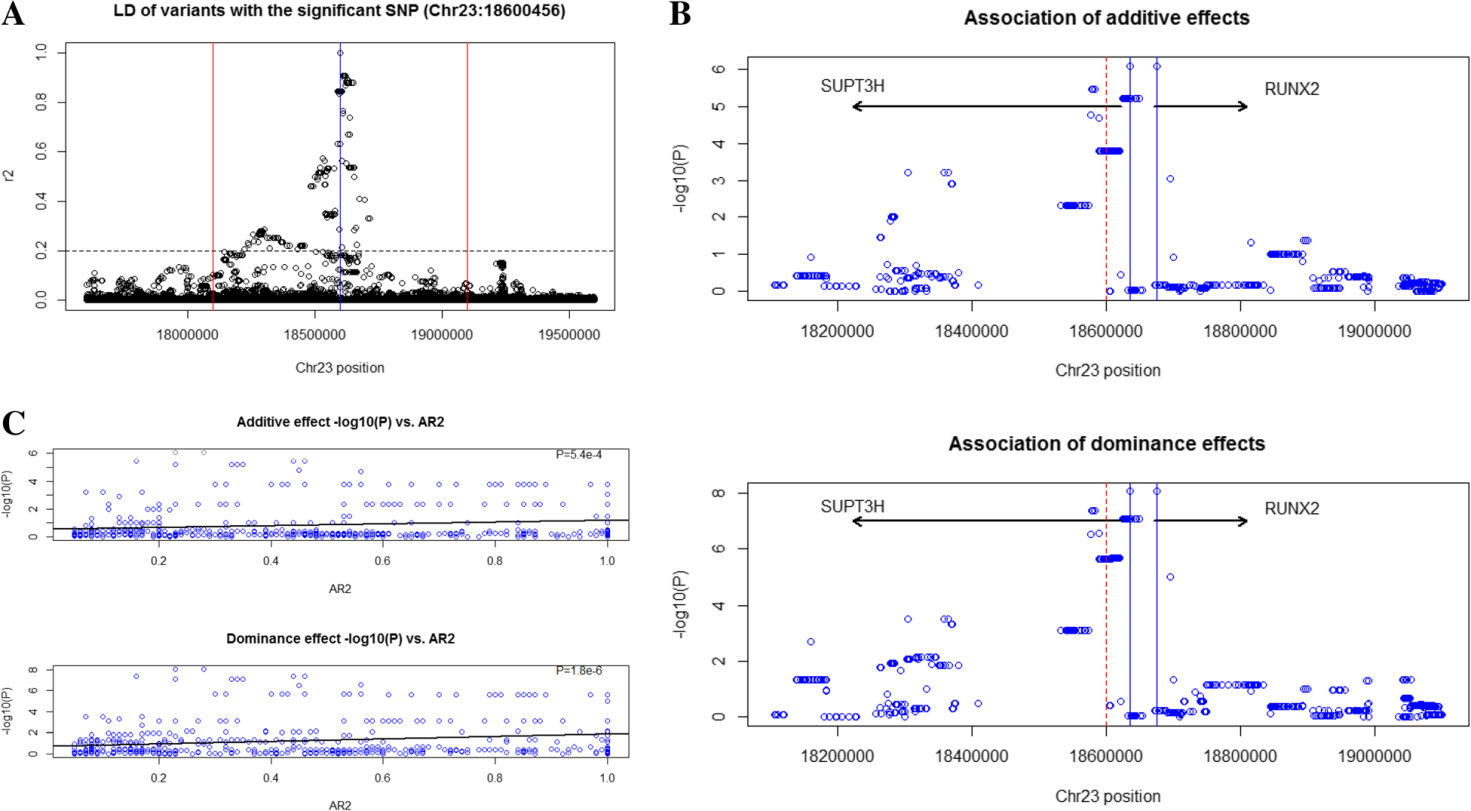
Fine-mapping of the dominance association with milk yield near *RUNX2*. **a** LD between BovineHD2300004730 and adjacent variants. **b** Association results of additive and dominance effects. The red dash line indicates the target SNP (BovineHD2300004730), while the two blue solid lines indicate the two variants with the smallest *P*-value. **c** The influence of imputation reliability measured by AR^2^ on association *P*-values. The black lines indicate the regression line of –log_10_(*P*) on AR^2^, and at the right-upper corner are the *P*-values for model fitting of the regression

From our GWAS and validation analyses, we selected BovineHD2300004730 (Chr23:18,600,456) as our target region for fine-mapping using sequence-based imputation. Based on the LD decay pattern between BovineHD2300004730 and nearby variants derived from the sequences of 443 Holstein bulls from the 1000 Bull Genomes project (Run 5.0) [24], we chose the region of ±500 kb from the targeted SNP for fine mapping to cover all the variants with a LD level of *r* ^2^ > 0.2 with BovineHD2300004730 (Fig. 5a). Using the 443 Holstein sequences as reference, we then imputed sequence-level SNPs in the targeted region for 29,811 cows. After post-imputation quality control (Additional file 7: Figure S3), a total of 652 variants were included in a two-step association analysis for milk yield.

The fine-mapping study identified 38 imputed variants with a stronger association than BovineHD2300004730 (Additional file 8: Table S5 and Fig. 5b). The smallest *P*-value for dominance effect (8.64 × 10^−9^) was found at two variants, one in the first intron of *RUNX2* (Chr23:18,676,057) and the other between *SUPT3H* and *RUNX2* (Fig. 5b). Although the 38 variants were all modifiers (Additional file 8: Table S5), the fine mapping analysis provided more evidence that the QTL is close to the *RUNX2* gene. Additionally, most of the variants had a larger dominance effect than additive effect, which was consistent with our original results supporting a dominant or over-dominant mode of inheritance. To investigate whether or not the significant associations were resulted from a single signal, we conducted a conditional analysis by adding the top variant (Chr23:18,676,057) as a covariate into the association test of each of the remaining 651 variants. This analysis revealed that the significant additive associations disappeared while the dominance signals remained (Additional file 9: Figure S4A). Conditioning on both the additive and the dominance effects eliminated all of the significant additive and dominance associations, indicating a single underlying QTL responsible for the association (Additional file 9: Figure S4B).

Since we imputed relatively low-density genotypes to sequence genotypes, imputation accuracy was a concern because poor imputation may result in smaller *P*-values in our fine-mapping analysis. We examined the impact of imputation accuracy (measured by AR^2^) on association *P*-values and found that poorly imputed variants tended to have a larger association *P*-value (Fig. 5c). This trend reduced the chance of getting false positives from low-quality imputation and provided additional support for the dominance association signal at *RUNX2* with milk yield.

### Genomic prediction incorporating dominance and imprinting effects

**Fig. 6:**
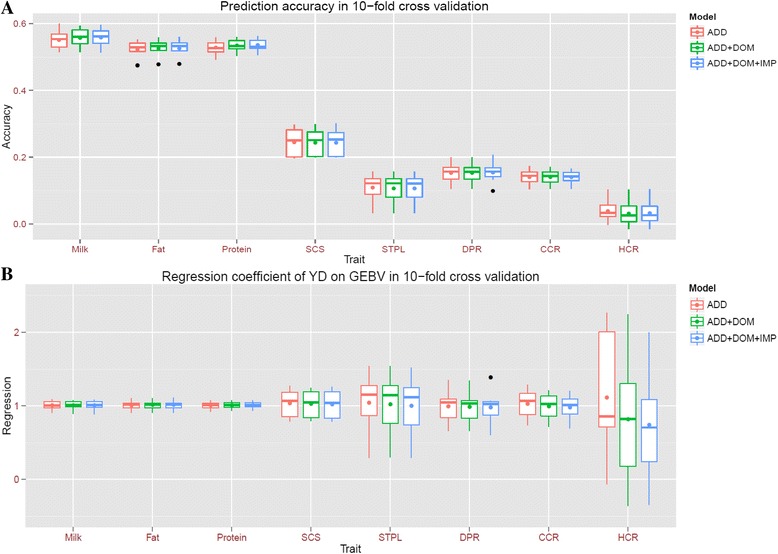
Prediction performance of three models for eight dairy traits. **a** Prediction accuracy in 10-fold cross validation. **b** Regression coefficient of YD on GEBV in 10-fold cross validation

We compared prediction performance of three models: 1) additive effect only (ADD), 2) additive and dominance effects (ADD + DOM), and 3) additive, dominance, and imprinting effects (ADD + DOM + IMP). Overall, the three models showed similar prediction accuracy and unbiasedness for all the eight traits (Fig. 6), even though non-additive effects explained >30% of total genetic variance for the three reproduction traits (DPR, CCR, and HCR). A small increase of prediction accuracy for three production traits (<1%) was observed with the models ADD + DOM and ADD + DOM + IMP compared to the model ADD. Paired t-tests showed that the increases were significant (*P* < 0.05). However, there was no significant difference in prediction accuracy between the models ADD + DOM and ADD + DOM + IMP for the three traits.

## Discussion

This study provided a systematic view of dominance and imprinting effects through a comprehensive analysis of a large cattle data set, including variance decomposition, GWAS, and genomic prediction. The study of imprinting effects benefited from the large size of the cattle data which included complete pedigree, representing one of the largest pedigrees available in a mammalian species, to infer parent-of-origin of alleles. The current study provided another demonstration of the power of dairy industry-oriented data to facilitate biological research [25, 26].

In general, our results are consistent with previous studies regarding the proportion of phenotypic variance explained by dominance effects for complex traits in cattle [10] and the low heritability of reproduction traits [27]. The U.S. national evaluation includes a regression on inbreeding to account for the effect of dominance on the mean, not just the variance and covariance. Sun et al. (2014) found a large advantage in predicting progeny performance by multiplying this regression on inbreeding by estimated genomic inbreeding of the calf, but found only small additional advantage by including dominance variance matrix. However, imprinting effects have been rarely evaluated in livestock studies, and our analysis provided useful information on the contribution of imprinting effects to dairy traits. First, despite their small proportion relative to the total variance, imprinting effects had a significant, non-zero contribution to the phenotypic variation for most of the traits investigated, including all the three production traits and three reproduction traits. Second, imprinting effects explained a much larger proportion of the total genetic variance for reproduction traits than for production traits. These results raised two important questions: does imprinting universally contribute to complex traits, and why are reproduction traits more affected by imprinting? It is worth mentioning that the reproduction traits considered here model pregnancy as a trait of the dam, whereas pregnancy as a trait of the embryo might have a stronger connection to dominance and imprinting.

In this study, we didn’t observe much improvement of prediction accuracy by including dominance and imprinting effects in genomic selection models. This observation can be attributable to a few things: 1) low heritability of non-additive effects; and 2) lacking of full-sib pairs between reference and prediction populations because full-sibs are the primary source of non-additive relationships but dairy data consist of mostly half-sibs.

Using a GWAS approach, we found a dominance association signal and validated it in independent samples. The fine-mapping analysis further confirmed the dominance QTL to be near *RUNX2*, but it was difficult to distinguish causal variants from linked markers. Due to a very small effective population size and a limited number of haplotypes in the dairy cattle population, our imputation works well, even from 50 k or less SNP data to sequence-level variants, in our fine-mapping association analysis.

Our study demonstrated the possibility of identifying non-additive effects in GWAS using a large dataset. Additionally, the power of the two-step GWAS approach was comparable to a full mixed-model based method (Table 1 and Additional file 5: Table S3). The two-step method used in this study was an efficient alternative to identify non-additive effects when fast implementations of full mixed-models are not available. For genomic prediction, we observed a very small but significant increase of prediction accuracy for production traits, but no difference for reproduction traits, when non-additive effects were included. Due to possible sparseness of dominance and imprinting effects, GREML may underperform for prediction and Bayesian models assuming a few large QTLs may perform better. Future studies are needed to develop more accurate prediction models for non-additive effects.

## Conclusions

In this study, we comprehensively evaluated the contribution of dominance and imprinting effects to complex traits in dairy cattle. We reported significant, non-zero contributions from dominance and imprinting effects for both production and reproduction traits. The imprinting effects contribute a larger proportion to reproduction traits that production traits. Using GWAS, we identified and validated a dominance association signal with milk yield near *RUNX2*. However, we observed minor increases in prediction accuracy when including non-additive effects in the genomic selection models.

## Methods

### Genotype and phenotype data

The large dairy cattle database maintained by CDCB and USDA-AGIL includes more than one million genotyped animals with complete pedigree. The data were collected on a continuous basis, and this study included all the Holstein data available until September, 2015. From the database, we extracted 262,757 genotyped females whose sire and dam were also genotyped. The genotypes were generated from 16 different SNP arrays with SNP number ranging from 7 K to 50 K. The SNP genotypes of all 262,757 females were phased to determine the parent-of-origin of each allele. We first used parent genotypes to phase a SNP genotype of a cow [26]. If this step failed, we then applied a population-based phasing approach using FindHap version 3.0 [28]. After phasing, all individuals were imputed to 50 K SNP data. When building genomic relationship matrices (GRMs), we further filled a small portion of genotypes that were still missing after imputation from FindHap by randomly sampling genotypes from a multinomial distribution with probabilities of the three genotypes derived under an assumption of Hardy–Weinberg equilibrium.

Among the 262,757 Holstein cows, 42,701 of them had yield deviation (YD) phenotypic data. YD phenotypes were adjusted for appropriate covariates, including farm, year, and season effects. Eight traits were analyzed, including milk yield (MY), fat yield (FY), protein yield (PY), somatic cell score (SCS; a measure of mammary gland health), standardized productive life (STPL; a measure of longevity), daughter pregnancy rate (DPR; a measure of fertility), cow conception rate (CCR; a measure of fertility), and heifer conception rate (HCR; a measure of fertility). Since many cows were not measured for all the phenotypes, the final sample size for the eight traits ranged from 12,911 (STPL) to 29,811 (MY, FY and PY), as shown in Table 1.

### Variance decomposition with additive, dominance, and imprinting components

Genetic effects of SNPs can be decomposed into three components (i.e., genotypic additive, dominance, and imprinting values), following their evident biological meanings:

1 $$ \left[\begin{array}{l}{G}_{11}\\ {}{G}_{12}\\ {}{G}_{21}\\ {}{G}_{22}\end{array}\right]= R+ a\left[\begin{array}{l}0\\ {}1\\ {}1\\ {}2\end{array}\right]+ d\left[\begin{array}{l}0\\ {}1\\ {}1\\ {}0\end{array}\right]+ i\left[\begin{array}{c}0\\ {}-1\\ {}1\\ {}0\end{array}\right]= R+ A+ D+ I, $$

where *G*_12_ is the genetic value for the genotype 12 with a paternal allele 1 and a maternal allele 2 (similar for *G*_11_, *G*_21_ and *G*_22_), *R* is the overall mean, *a* is additive effect, *d* is dominance effect, *i* is imprinting effect, *A* is genotypic additive value arising from *a*, *D* is genotypic dominance value arising from *d*, and *I* is genotypic imprinting value arising from *i*. Under Hardy-Weinberg equilibrium, eq. (2) can be further centralized regarding *a* and *d* into

2 $$ \left[\begin{array}{l}{G}_{11}\\ {}{G}_{12}\\ {}{G}_{21}\\ {}{G}_{22}\end{array}\right]={R}^{\ast }+ a\left[\begin{array}{c}-2 p\\ {} q- p\\ {} q- p\\ {}2 q\end{array}\right]+ d\left[\begin{array}{c}-2 p q\\ {}1-2 p q\\ {}1-2 p q\\ {}-2 p q\end{array}\right]+ i\left[\begin{array}{c}0\\ {}-1\\ {}1\\ {}0\end{array}\right]={R}^{\ast }+{A}^{\ast }+{D}^{\ast }+ I, $$

where *R* ^*^ is the overall mean after centering, *p* is the frequency of allele 2 and *q* is the frequency of allele 1, and *A* ^*^ (*D* ^*^) is genotypic additive (dominance) value after centralization. Note that in eq. (2), genotypic additive value (*A* ^*^) is not independent of genotypic dominance value (*D* ^*^), or *Cov*(*A* ^∗^, *D* ^∗^) ≠ 0. To address the issue, we can use the extended natural and orthogonal interactions (NOIA) model [21] under Hardy-Weinberg equilibrium,

3 $$ \left[\begin{array}{l}{G}_{11}\\ {}{G}_{12}\\ {}{G}_{21}\\ {}{G}_{22}\end{array}\right]={R}^{\ast \ast }+\beta \left[\begin{array}{c}-2 p\\ {} q- p\\ {} q- p\\ {}2 q\end{array}\right]+ d\left[\begin{array}{c}-2{p}^2\\ {}2 p q\\ {}2 p q\\ {}-2{q}^2\end{array}\right]+ i\left[\begin{array}{c}0\\ {}-1\\ {}1\\ {}0\end{array}\right]={R}^{\ast \ast }+{A}^{\ast \ast }+{D}^{\ast \ast }+ I, $$

where *R* ^**^ is the overall mean and *β* is allele substitution effect. Despite its similarity to eq. (2), eq. (3) results in different variance decomposition. The three components for *β*, *d*, and *i* correspond to breeding value (*A* ^**^), dominance deviation (*D* ^**^), and genotypic imprinting value (*I*), respectively.

The differences and relationships between eqs. (2) and (3) have been thoroughly discussed in a previous study [6], although that study did not include imprinting effects. The equation still holds when imprinting effects are included because the genotypic imprinting value is independent of the other two components in both eqs. (2) and (3). In theory, the sum of individual breeding value and dominance deviation in eq. (3) is equal to the sum of individual genotypic additive and dominance values in eq. (2); and when ignoring the covariance between additive and dominance effects, the sum of additive and dominance genetic variances resulting from the decomposition by eq. (3) is equal to the sum of the variances of genotypic additive and dominance values resulting from the decomposition by eq. (2). Additionally, individual genotypic imprinting value in eq. (2) is the same as in eq. (3), thus asserting the equivalence of imprinting variance components in the two equations. The theory holds for multiple loci when assuming linkage equilibrium and independent marker effects [6].

Although it is possible to directly fit SNP effects in a model [29], fitting individual-level genetic components is more efficient, especially for a large dataset with many SNP markers. In this study, we used the following model

4 $$ \mathbf{y}=\mathbf{Xb}+\mathbf{a}+\mathbf{d}+\mathbf{i}+\mathbf{e}\kern0.5em \mathrm{with}\ \mathbf{a}\sim N\left(0,{\sigma}_a^2\mathbf{A}\right),\mathbf{d}\sim N\left(0,{\sigma}_d^2\mathbf{D}\right),\mathbf{i}\sim N\left(0,{\sigma}_p^2\mathbf{P}\right),\mathbf{e}\sim N\left(0,{\sigma}_e^2\mathbf{I}\right), $$

where the phenotypic value of individuals (***y***) was decomposed into fixed effects (***b***), genotypic additive value (***a***), genotypic dominance value (***d***), genotypic imprinting value (***i***), and residual (***e***). Eq. (4) can be readily solved by a multi-component restricted maximum likelihood (REML) approach as implemented in GCTA [30], as long as we know the covariance structures of the three components, **A**, **D**, and **P**. Different forms of additive genomic relationship matrix (GRM) have been proposed. We used a version with pooled variance across all markers [31],

5 $$ {A}_{ij}={\sum}_k\left({Z}_{ik}-2{p}_k\right)\left({Z}_{jk}-2{p}_k\right)/{\sum}_k2{p}_k\left(1-{p}_k\right), $$

where *Z*_*ik*_ (*Z*_*jk*_) is the additive genotype code for marker *k* of individual *i* (*j*) as shown in the vector corresponding to *a* in eq. (1) and *p*_*k*_ is the population frequency of allele 2. Similarly, based on the equivalence of SNP-BLUP and GBLUP [7, 32], we can obtain corresponding GRMs for dominance (**D**) and imprinting (**P**), which are shown as following:

6 $$ {D}_{ij}={\sum}_k\left[{H}_{ik}-2{p}_k\left(1-{p}_k\right)\right]\left[{H}_{jk}-2{p}_k\left(1-{p}_k\right)\right]/{\sum}_k2{p}_k\left(1-{p}_k\right)\left[1-2{p}_k\left(1-{p}_k\right)\right] $$

7 $$ {P}_{ij}={\sum}_k{S}_{ik}{S}_{jk}/{\sum}_k2{p}_k\left(1-{p}_k\right) $$

where *H* and *S* are the genotype codes for dominance and imprinting effects as shown in the corresponding vectors in eq. (1), respectively. Equation (6) has been used in previous studies [5, 10]. When building GRMs, we used whole-genome markers with minor allele frequency (MAF) ≥0.01. Finally, the software MMAP [33], which efficiently implements REML, was used to fit model (4).

For comparison purposes, we also performed variance decomposition based on eq. (3). In this case, we need to use a different dominance GRM (**D** ^*^),

8 $$ {D}_{ij}^{\ast }={\sum}_k{H}_{ik}^{\ast }{H}_{jk}^{\ast }/{\sum}_k{\left(2{p}_k\left(1-{p}_k\right)\right)}^2, $$

where H* is the dominance genotype code as shown in the vector corresponding to *d* in eq. (3). Accordingly, the total genetic variance is decomposed to classical additive and dominance genetic variances and variance of genotypic imprinting effect. We further compared the two different kinds of variance decompositions regarding estimates of individual effects and variance components to verify the theory on their equivalence of explaining phenotypes.

### Simulation study for validating variance decomposition

Note that when building the GRMs, we assumed that the traits are highly polygenic for the additive, dominance, and imprinting effects. Although the polygenic architecture of additive effects is commonly used for complex traits [22], we have less knowledge on whether dominance and imprinting effects are also polygenic. To address this issue, we simulated a number of datasets to investigate whether model (4) can capture dominance and imprinting effects when there are a small number of corresponding QTLs. Specifically, we first obtained a random subsample of 10,000 from the 42,000 cows being analyzed, and then randomly selected markers from the 50 k SNPs as additive, dominance, or imprinting QTLs. We simulated QTL effects using a normal distribution and added them up to obtain *a*, *d*, and *i* for each of the 10,000 cows. Thereafter we calculated ${\sigma}_a^2=\operatorname{var}(a)$, ${\sigma}_d^2=\operatorname{var}(d)$, and ${\sigma}_p^2=\operatorname{var}(i)$ using corresponding simulated genetic values. Based on the heritability we set to simulate, we calculated ${\sigma}_e^2$ and simulated *e* by sampling it from $N\left(0,{\sigma}_e^2\right)$. The phenotype for each individual animal was simulated by adding up *a*, *d*, *i*, and *e*.

To ensure realistic simulations, we picked variance of the normal distribution for simulating effect sizes so the variance decomposition was the same between simulated and real data. Our simulation scenarios included two representative traits, milk yield and DPR, separately. Three scenarios were simulated for either trait by varying QTL numbers, including 1000 + 10 + 10 (1000, 10 and 10 QTLs for additive, dominance, and imprinting effects, respectively), 1000 + 100 + 100, and 1000 + 1000 + 1000. Simulation for each scenario was repeated 100 times. We fitted model (4) for each simulated data set and compared variance component estimation between the three scenarios.

### Genome-wide association study of non-additive effects

To increase computational efficiency, we used a two-step strategy for genome-wide association study, similar to the GRAMMAR approach [34]. First, we fitted model (4), and obtained the residuals to adjust for polygenic effects. Second, we used the residuals as response variable to fit a multiple linear regression model for each SNP,

9 $$ \mathbf{e}=\mu +{a}_k{\mathbf{Z}}_k+{d}_k{\mathbf{H}}_k+{i}_k{\mathbf{S}}_k+\boldsymbol{\upvarepsilon}, $$

where **Z**_*k*_, **H**_*k*_ and **S**_*k*_ are the genotype codes of marker *k* for additive, dominance and imprinting effects, respectively, as described in eqs. (5,6,7), and *a*_*k*_, *d*_*k*_, and *i*_*k*_ are corresponding SNP effects. SNPs were filtered by MAF ≥0.01 and *P*-value of Chi-square test for Hardy–Weinberg equilibrium ≥1 × 10^−6^. Association *P*-values were calculated from t-tests for the three types of SNP effects.

For association signals with sufficient statistical evidence from the two-step analysis, we further used the full mixed model,

10 $$ \begin{array}{c}\mathbf{y}=\mu +{a}_k{\mathbf{Z}}_k+{d}_k{\mathbf{H}}_k+{i}_k{\mathbf{S}}_k+\mathbf{a}+\mathbf{d}+\mathbf{i}+\mathbf{e}\\ {}\mathrm{with}\ \mathbf{a}\sim N\left(0,{\sigma}_a^2\mathbf{A}\right),\mathbf{d}\sim N\left(0,{\sigma}_d^2\mathbf{D}\right),\mathbf{i}\sim N\left(0,{\sigma}_p^2\mathbf{P}\right),\mathbf{e}\sim N\left(0,{\sigma}_e^2\mathbf{I}\right),\end{array} $$

or its reduced version,

11 $$ \mathbf{y}=\mu +{a}_k{\mathbf{Z}}_k+{d}_k{\mathbf{H}}_k+{i}_k{\mathbf{S}}_k+\mathbf{a}+\mathbf{e}\kern0.5em \mathrm{with}\ \mathbf{a}\sim N\left(0,{\sigma}_a^2\mathbf{A}\right)\ \mathrm{and}\ \mathbf{e}\sim N\left(0,{\sigma}_e^2\mathbf{I}\right), $$

to rerun the association analysis, depending on whether the additive effects can explain a majority of total genetic variance on the trait being analyzed. Here, the response variables in eq. (10) and (11) are yield deviations. Again, we applied the software MMAP [33] to fit the mixed models.

### Validation of non-additive association signals using independent data

Our discovery GWAS used the data available until September, 2015. From then to April, 2016, we assembled a new dataset to validate the signal found in the initial GWAS. The validation data consisted of 5514 cows with both genotypes and milk phenotypes. The genotypes in the validation data were phased with the same procedures as used for the discovery data set. With the validation data, model (11) was used to analyze associations between milk and 50 SNP markers around the *RUNX2* signal. The GRM was built using all chip SNPs except those on chromosome 23, which resulted in a leave-one-chromosome-out analysis (LOCO) [35]. We also built the GRM using all genome-wide SNPs and compared it with the LOCO analysis. The validation data were also used to analyze the significant dominance associations around Chr5:107,000,000 with both fat and protein. The three SNPs with the smallest discovery *P*-value were analyzed with model (11) for fat and protein, respectively.

### Fine mapping for the *RUNX2* dominance signal

First, we used the sequence data of 443 Holstein bulls from the 1000 Bull Genomes project [24] (Run 5.0) to check LD levels between the targeted SNP (Chr23:18,600,456) and SNPs/biallelic indels around it. Based on the LD decay pattern, we chose the region of ±500 kb from the targeted SNP for fine mapping. Then, we used the sequence genotypes of the 443 bulls as reference to impute the 50 k genotypes of 29,811 cows to sequence genotypes. Beagle version 4 [36] was used for the imputation with default parameters. To increase accuracy, our imputation covered a larger region of ±1 Mb from the targeted SNP. After imputation, we removed non-informative SNPs, i.e. SNPs with a MAF <0.01, SNPs with a *P*-value of Chi-square test for Hardy–Weinberg equilibrium <1 × 10^−6^ and SNPs with an allelic *R* ^2^ (AR^2^) <0.05. AR^2^, reported by Beagle software, is the estimated squared correlation between the most probable alternative allele dose and the true alternative allele dose and serves as a good metric for estimating imputation accuracy [37]. The analysis of associations between milk and the imputed sequence variants within the targeted region (Chr23:18,100,456–19,100,456) was performed with a two-step method as described in our GWAS section.

### Genomic prediction

We estimated the values of the three effects for individuals in the training population from fitting model (4) in MMAP. The genomic predictions for new individuals can be calculated by

12 $$ {\widehat{\mathbf{g}}}_n={\widehat{\boldsymbol{\upalpha}}}_n+{\widehat{\mathbf{d}}}_n+{\widehat{\mathbf{i}}}_n={\mathbf{A}}_{n\times t}{\mathbf{A}}_{t\times t}^{-1}{\widehat{\mathbf{a}}}_t+{\mathbf{D}}_{n\times t}{\mathbf{D}}_{t\times t}^{-1}{\widehat{\mathbf{d}}}_t+{\mathbf{P}}_{n\times t}{\mathbf{P}}_{n\times t}^{-1}\widehat{{\mathbf{i}}_t} $$

where the subscripts *n* and *t* indicate the sets of new individuals and training population, respectively. Besides model (4) (ADD + DOM + IMP), we also considered two reduced models, the additive model (ADD) and the additive-plus-dominance model (ADD + DOM), and compared the prediction performance between the three models. Ten-fold cross validation was used to assess 1) prediction accuracy, defined as the Person correlation between genomic estimated breeding value (GEBV) and phenotype, and 2) unbiasedness, defined as the regression coefficient of phenotype on GEBV in the validation population.

## Additional files

Additional file 1: Table S1. Variance decomposition of breeding value, dominance deviation, and genotypic imprinting value for eight dairy traits. (XLSX 10 kb)

Additional file 2: Figure S1. Individual estimates of different components with two variance decomposition models for fat, protein, SCS, STPL, DPR, CCR and HCR. (PDF 115 kb)

Additional file 3: Table S2. Mixed-model association statistics of 50 SNPs around the *RUNX2* gene in the validation data set. (XLSX 17 kb)

Additional file 4: Figure S2. Manhattan plots for associations of SNP effects with fat, protein, SCS, STPL, DPR, CCR and HCR. (PDF 6350 kb)

Additional file 5: Table S3. Two-step and full model analysis results for candidate non-additive associations in the discovery data set. (XLSX 17 kb)

Additional file 6: Table S4. Full mixed model analysis results for the dominance associations with fat and protein in the validation data set. (XLSX 10 kb)

Additional file 7: Figure S3. AR^2^ of the sequence based imputation. (PDF 5 kb)

Additional file 8: Table S5. A full list of imputed sequence variants showing stronger association with milk than BovineHD2300004730. (XLSX 14 kb)

Additional file 9: Figure S4. Association analysis conditional on the additive effect (A) and both the additive and dominance effects (B) of variant Chr23:18,676,057. The vertical blue line indicates the location of Chr23:18,676,057. (PDF 14 kb)

## Abbreviations

CCR: Cow conception rate
Chr: Chromosome
DPR: Daughter pregnancy rate
FY: Fat yield
GEBV: Genomic estimated breeding value
GRM: Genomic relationship matrix
GWAS: Genome-wide association study
HCR: Heifer conception rate
Kb: Kilo base pairs = 1000 base pairs
LD: Linkage disequilibrium
Mb: Mega bases pairs = 1000 kb = 1 million base pairs
MY: Milk yield
PY: Protein yield
QTL: Quantitative trait locus
SCS: Somatic cell score
SNP: Single nucleotide polymorphism
STPL: Standardized productive life
YD: Yield deviatoin
