# Supplementary material for: Dissection of additive, dominance, and imprinting effects for production and reproduction traits in Holstein cattle
Source: BMC Genomics. 2017 May 30;18:425. doi: 10.1186/s12864-017-3821-4 (PMC5450346; doi:10.1186/s12864-017-3821-4)
Supplement: Supplementary file 2 — Individual estimates of different components with two variance decomposition models for fat, protein, SCS, STPL, DPR, CCR and HCR. (PDF 115 kb) [file 12864_2017_3821_MOESM2_ESM.pdf]

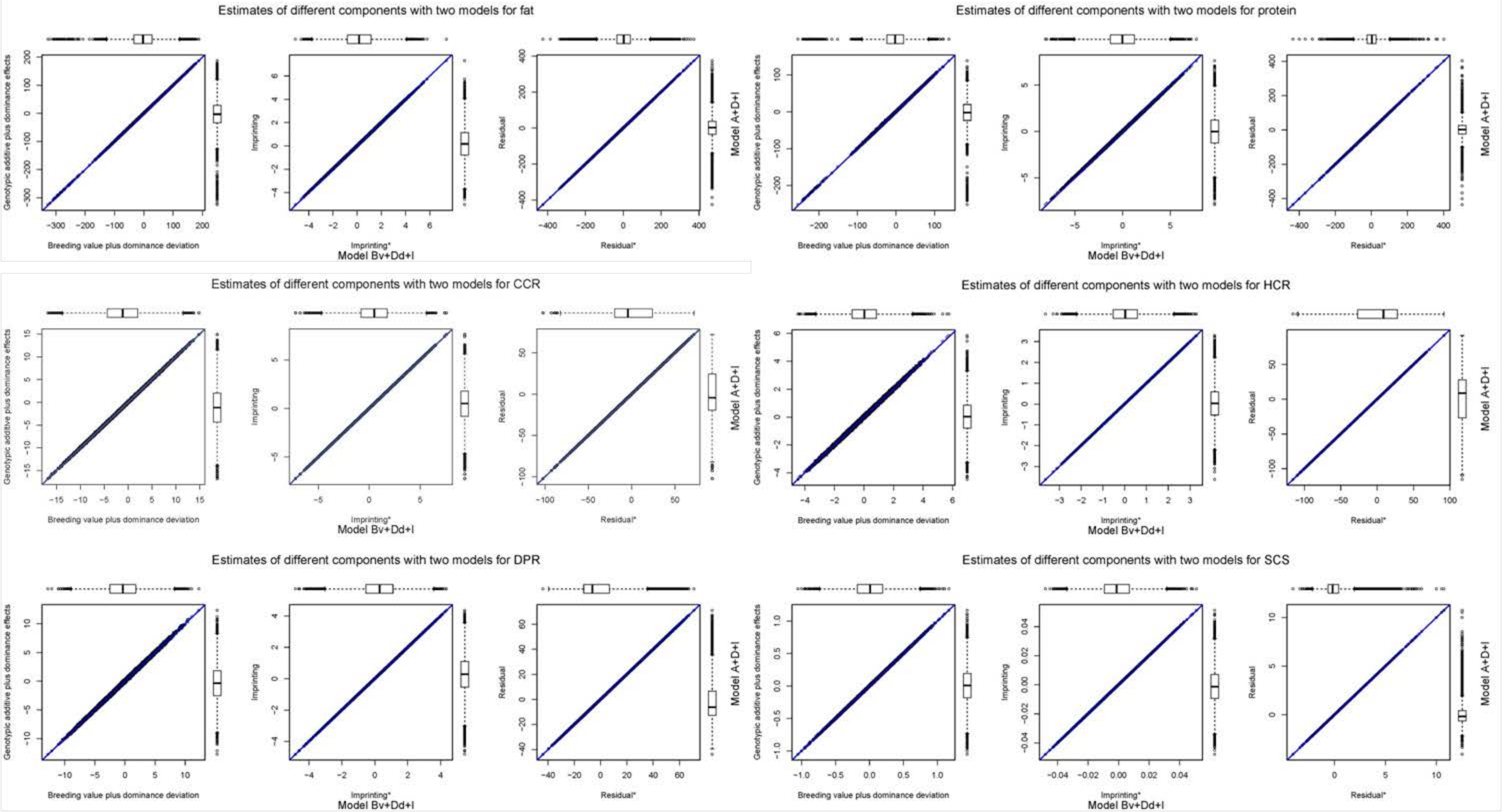
Estimates of different components with two models for fat
Estimates of different components with two models for protein
Estimates of different components with two models for CCR
Estimates of different components with two models for HCR
Estimates of different components with two models for DPR
Estimates of different components with two models for SCS
Genotypic additive plus dominance effects
Imprinting
Residual
Breeding value plus dominance deviation
Imprinting*
Residual*
Model Bv+Dd+I
Model A+D+I
