# Supplementary material for: Dissection of additive, dominance, and imprinting effects for production and reproduction traits in Holstein cattle
Source: BMC Genomics. 2017 May 30;18:425. doi: 10.1186/s12864-017-3821-4 (PMC5450346; doi:10.1186/s12864-017-3821-4)

Manhattan plots for association of SNPs with Fat

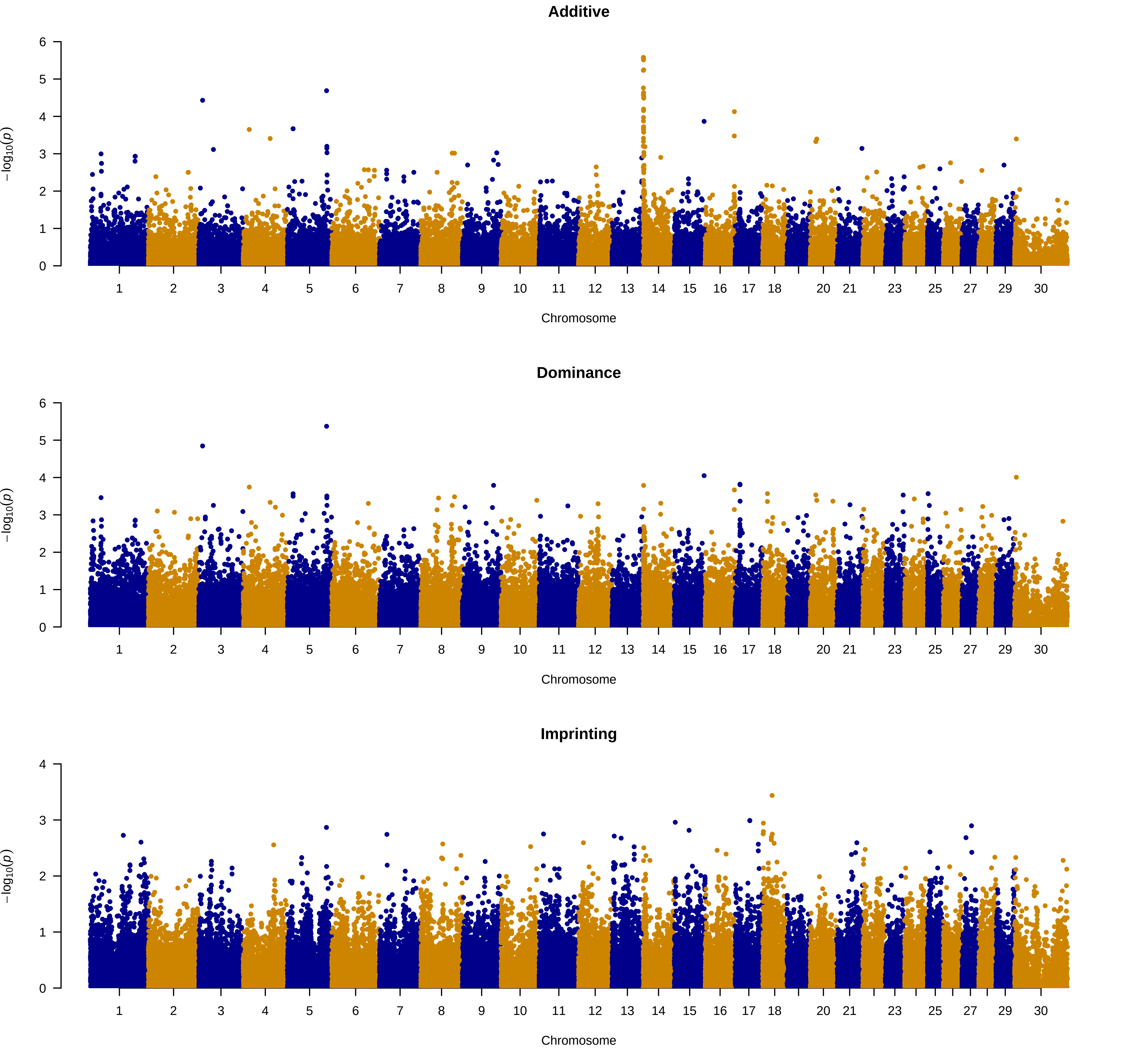

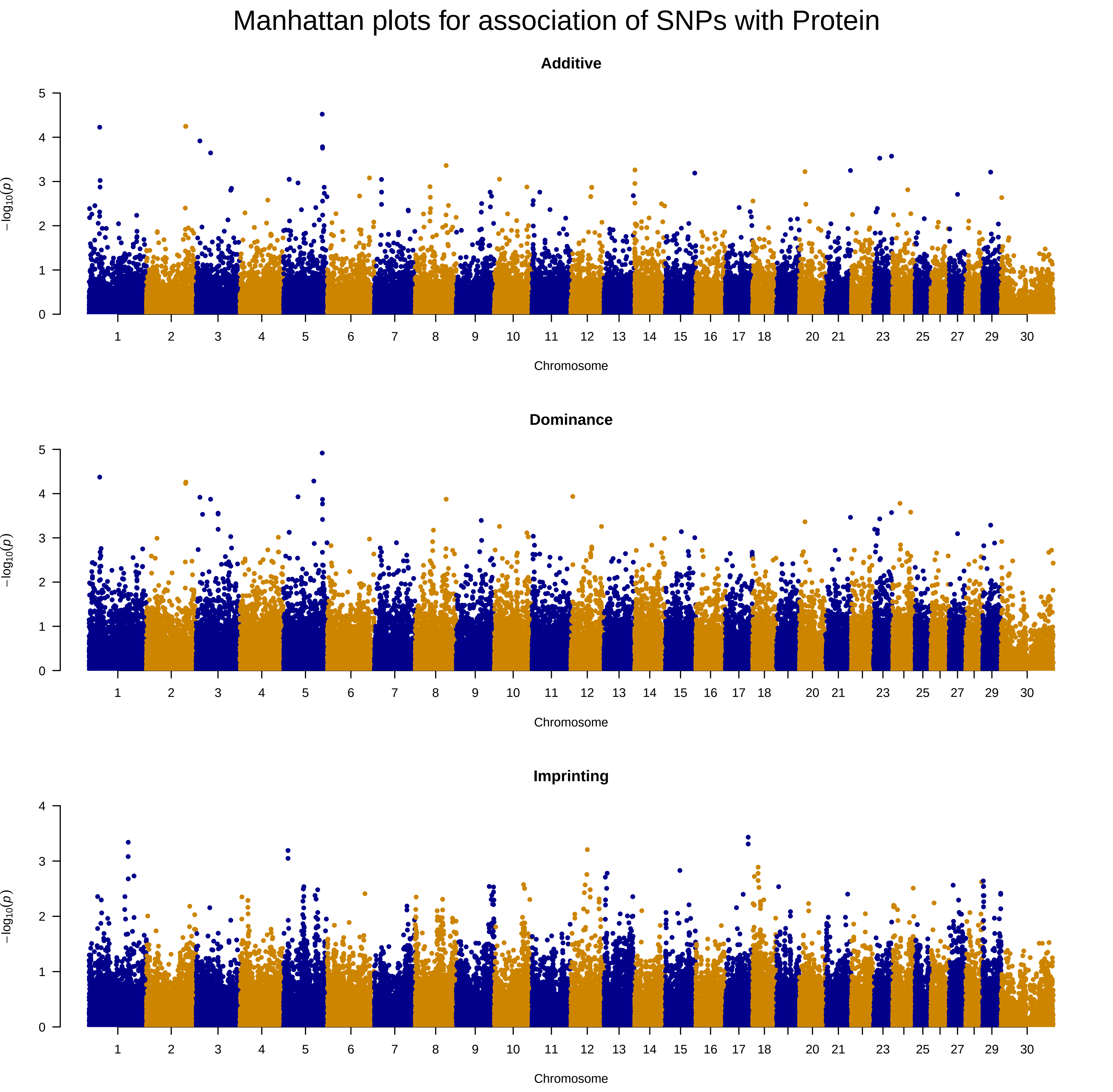
Manhattan plots for association of SNPs with Protein
Additive
Dominance
Imprinting
$-\log_{10}(p)$
Chromosome
1 2 3 4 5 6 7 8 9 10 11 12 13 14 15 16 17 18 20 21 23 25 27 29 30
0 1 2 3 4 5

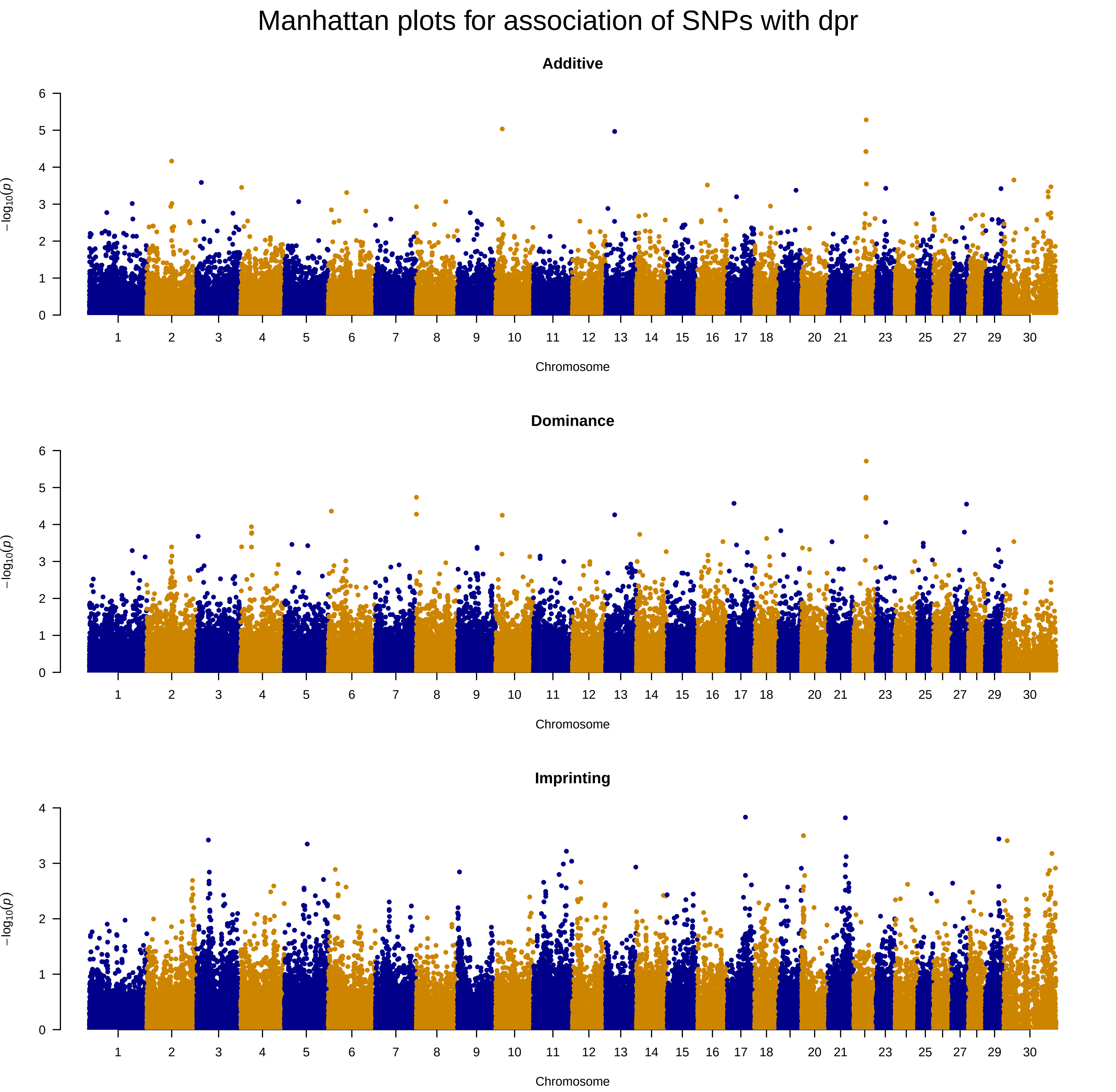
Manhattan plots for association of SNPs with dpr
Additive
Dominance
Imprinting
−log10(p)
Chromosome
1 2 3 4 5 6 7 8 9 10 11 12 13 14 15 16 17 18 20 21 23 25 27 29 30
0 1 2 3 4 5 6

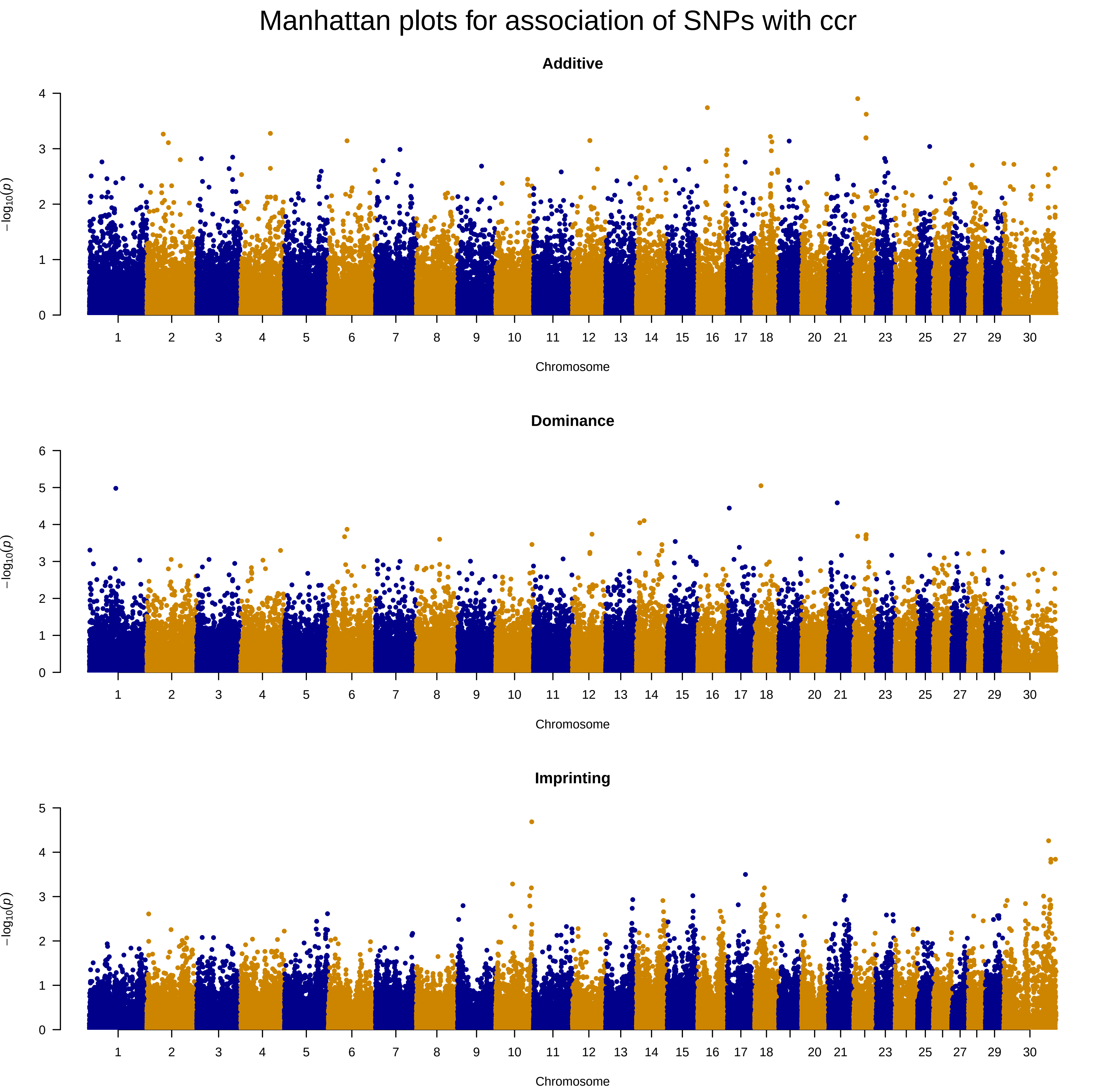
Manhattan plots for association of SNPs with ccr
Additive
Dominance
Imprinting
$-\log_{10}(p)$
Chromosome
1 2 3 4 5 6 7 8 9 10 11 12 13 14 15 16 17 18 20 21 23 25 27 29 30

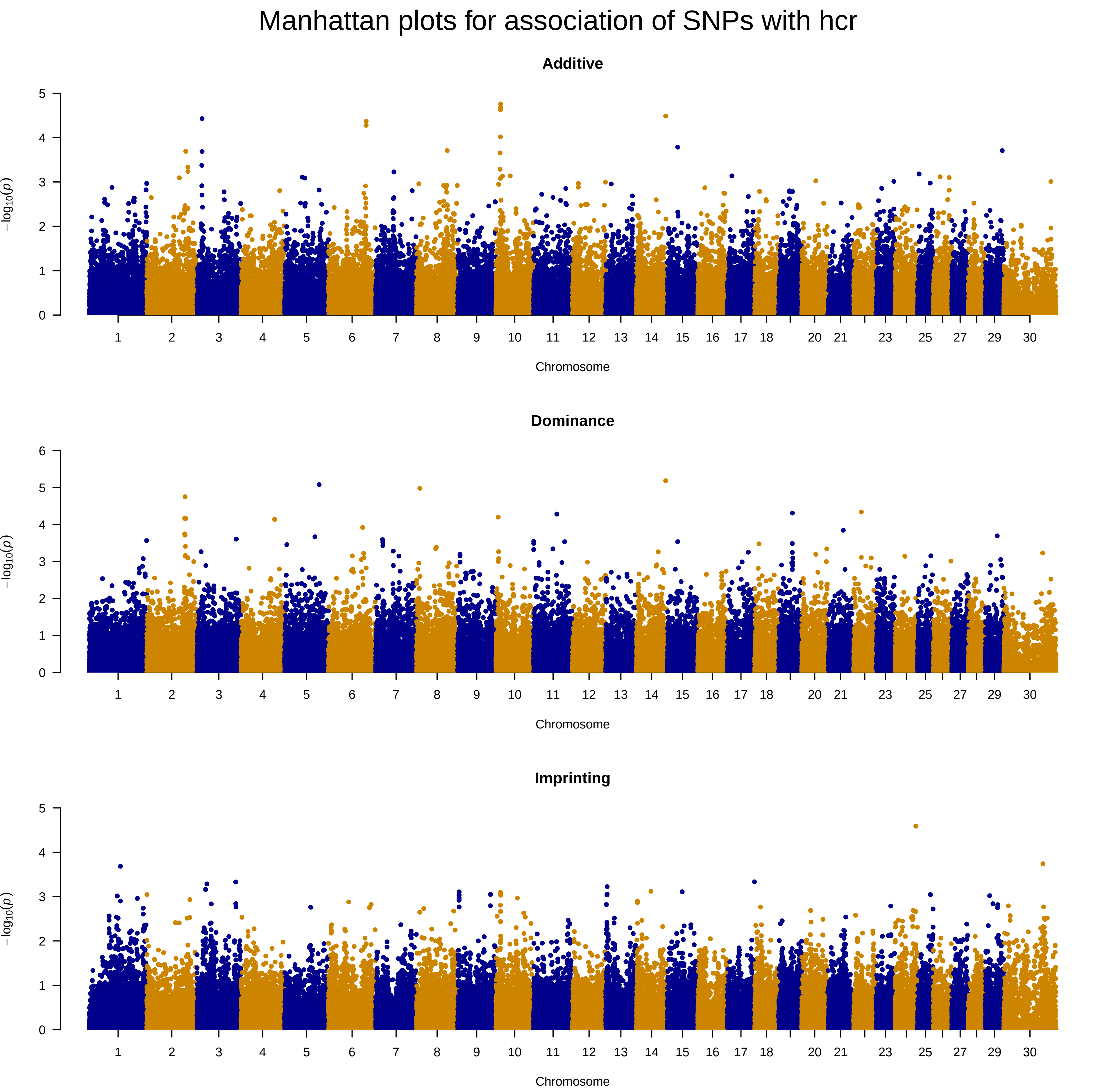
Manhattan plots for association of SNPs with hcr
Additive
Dominance
Imprinting
−log10(p)
Chromosome
0
1
2
3
4
5
6
1
2
3
4
5
6
7
8
9
10
11
12
13
14
15
16
17
18
20
21
23
25
27
29
30

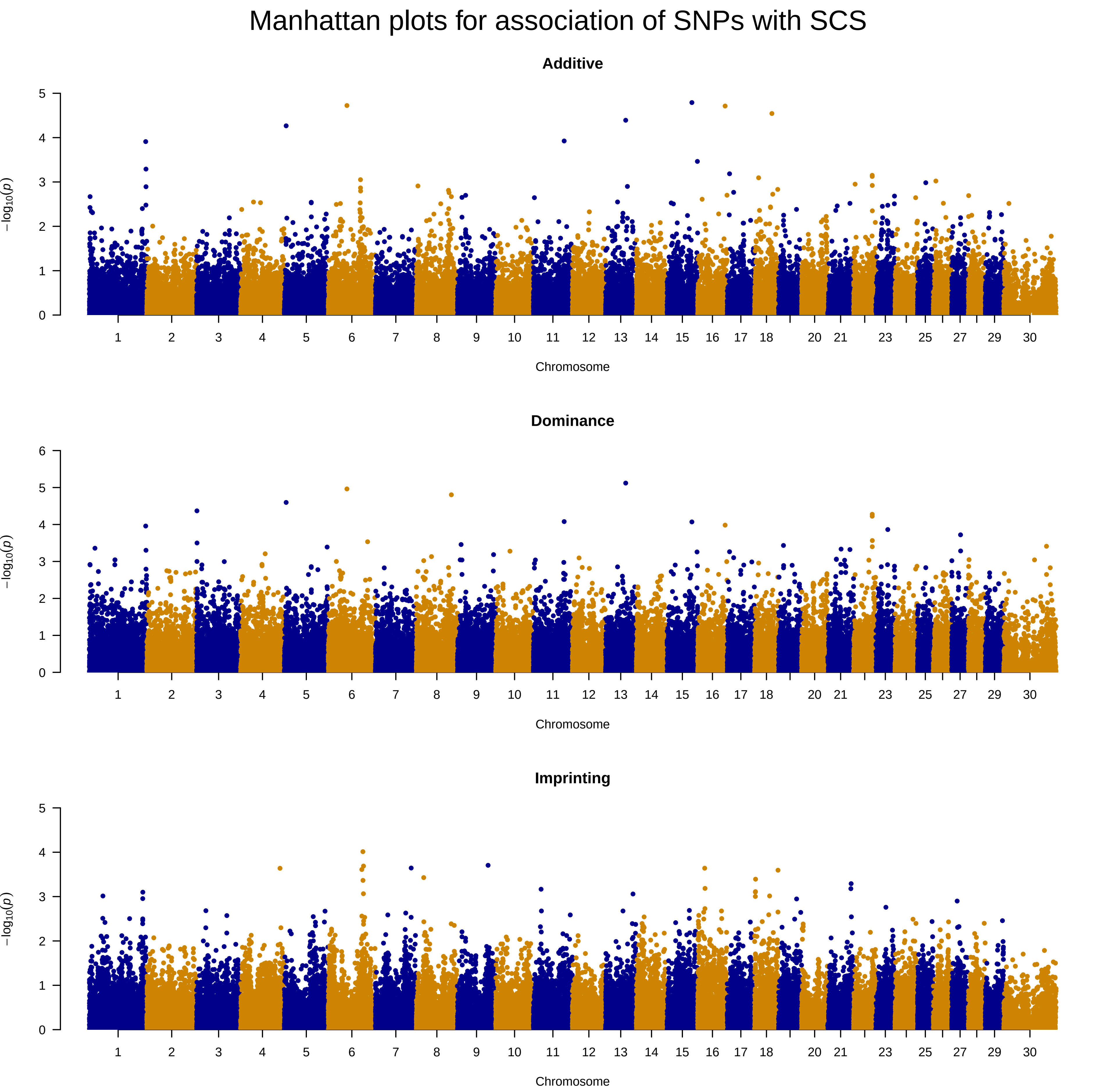
Manhattan plots for association of SNPs with SCS
Additive
Dominance
Imprinting
$-\log_{10}(p)$
Chromosome
0
1
2
3
4
5
6
7
8
9
10
11
12
13
14
15
16
17
18
20
21
23
25
27
29
30

Manhattan plots for association of SNPs with STPL

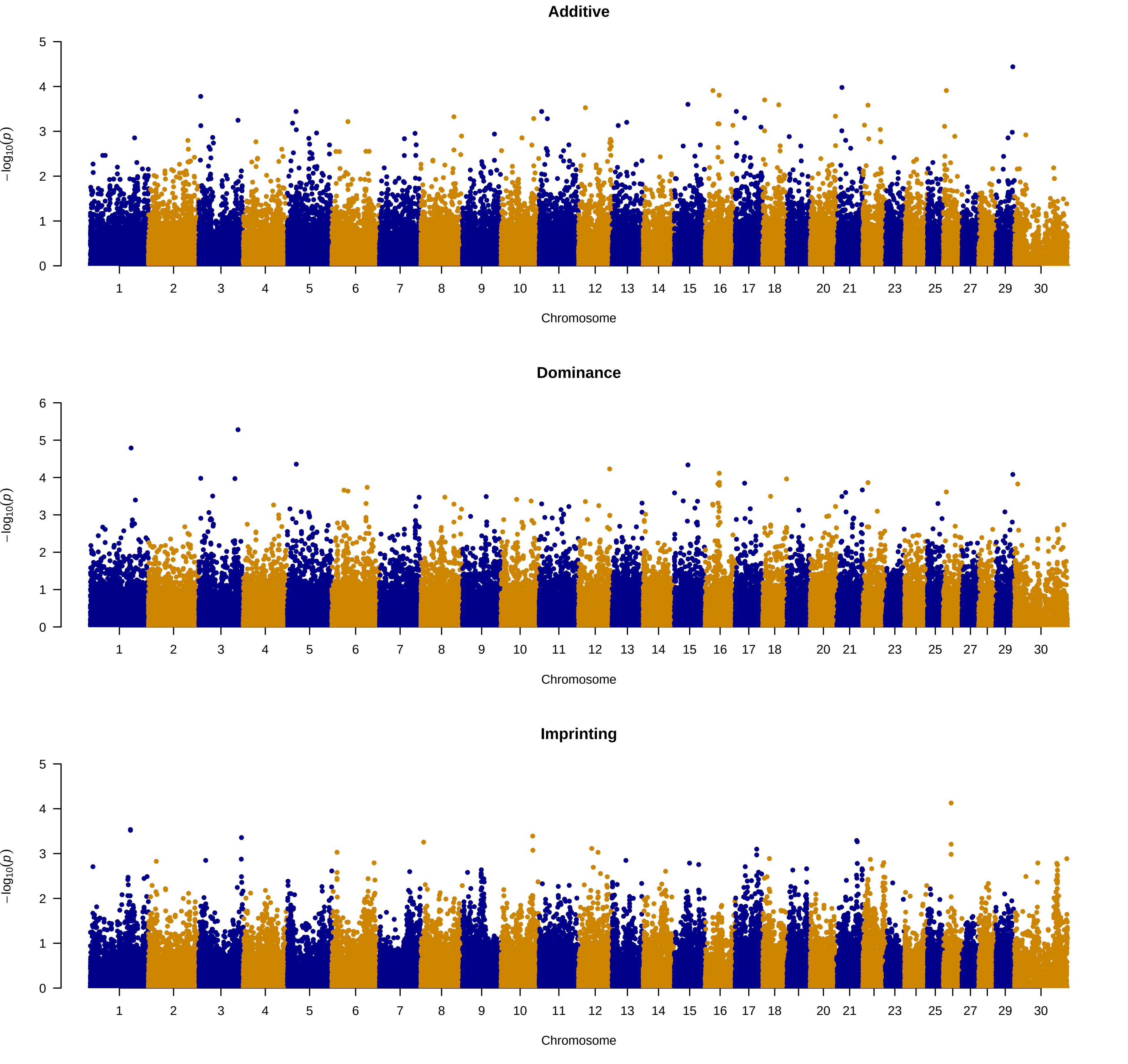

Supplement: Supplementary file 4 — Manhattan plots for associations of SNP effects with fat, protein, SCS, STPL, DPR, CCR and HCR. (PDF 6350 kb) [file 12864_2017_3821_MOESM4_ESM.pdf]
