# Supplementary figures and images for: Dissection of additive, dominance, and imprinting effects for production and reproduction traits in Holstein cattle
Source: BMC Genomics. 2017 May 30;18:425. doi: 10.1186/s12864-017-3821-4 (PMC5450346; doi:10.1186/s12864-017-3821-4)

### AR2 of the imputation around RUNX2

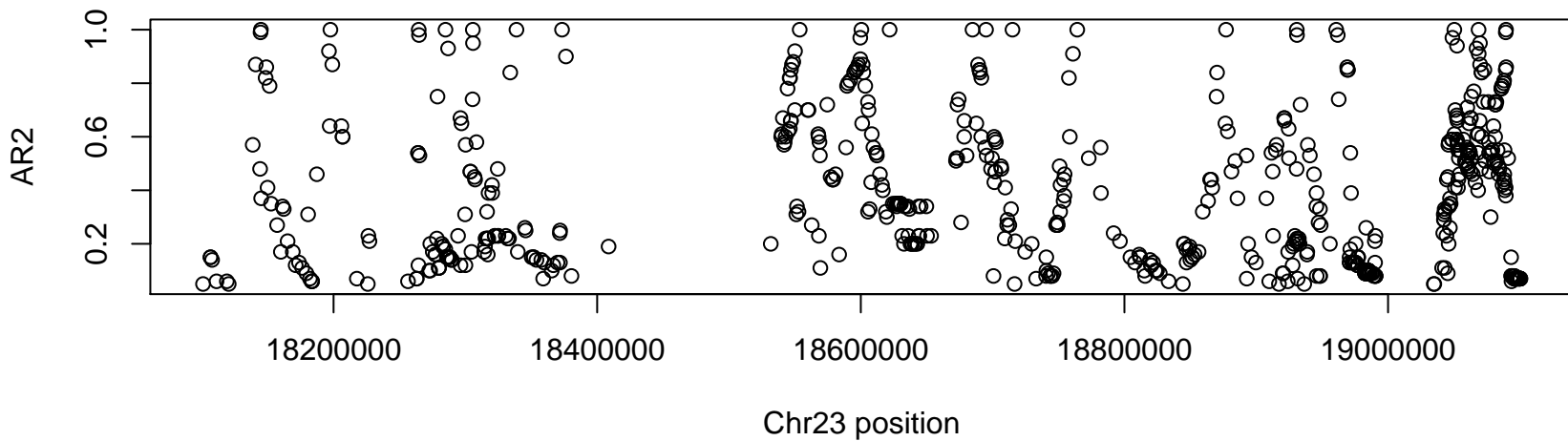

### AR2 distribution of the sequence based imputation

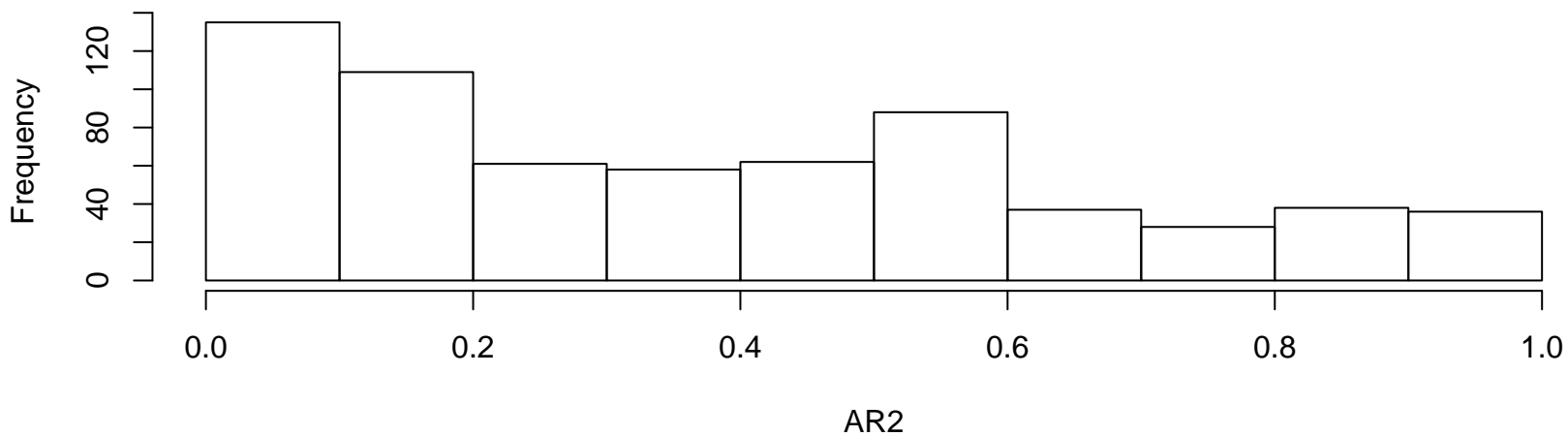

Supplement: Supplementary file 7 — AR2 of the sequence based imputation. (PDF 5 kb) [file 12864_2017_3821_MOESM7_ESM.pdf]
