# Supplementary material for: Dissection of additive, dominance, and imprinting effects for production and reproduction traits in Holstein cattle
Source: BMC Genomics. 2017 May 30;18:425. doi: 10.1186/s12864-017-3821-4 (PMC5450346; doi:10.1186/s12864-017-3821-4)
Supplement: Supplementary file 9 — Association analysis conditional on the additive effect (A) and both the additive and dominance effects (B) of variant Chr23:18,676,057. The vertical blue line indicates the location of Chr23:18,676,057. (PDF 14 kb) [file 12864_2017_3821_MOESM9_ESM.pdf]

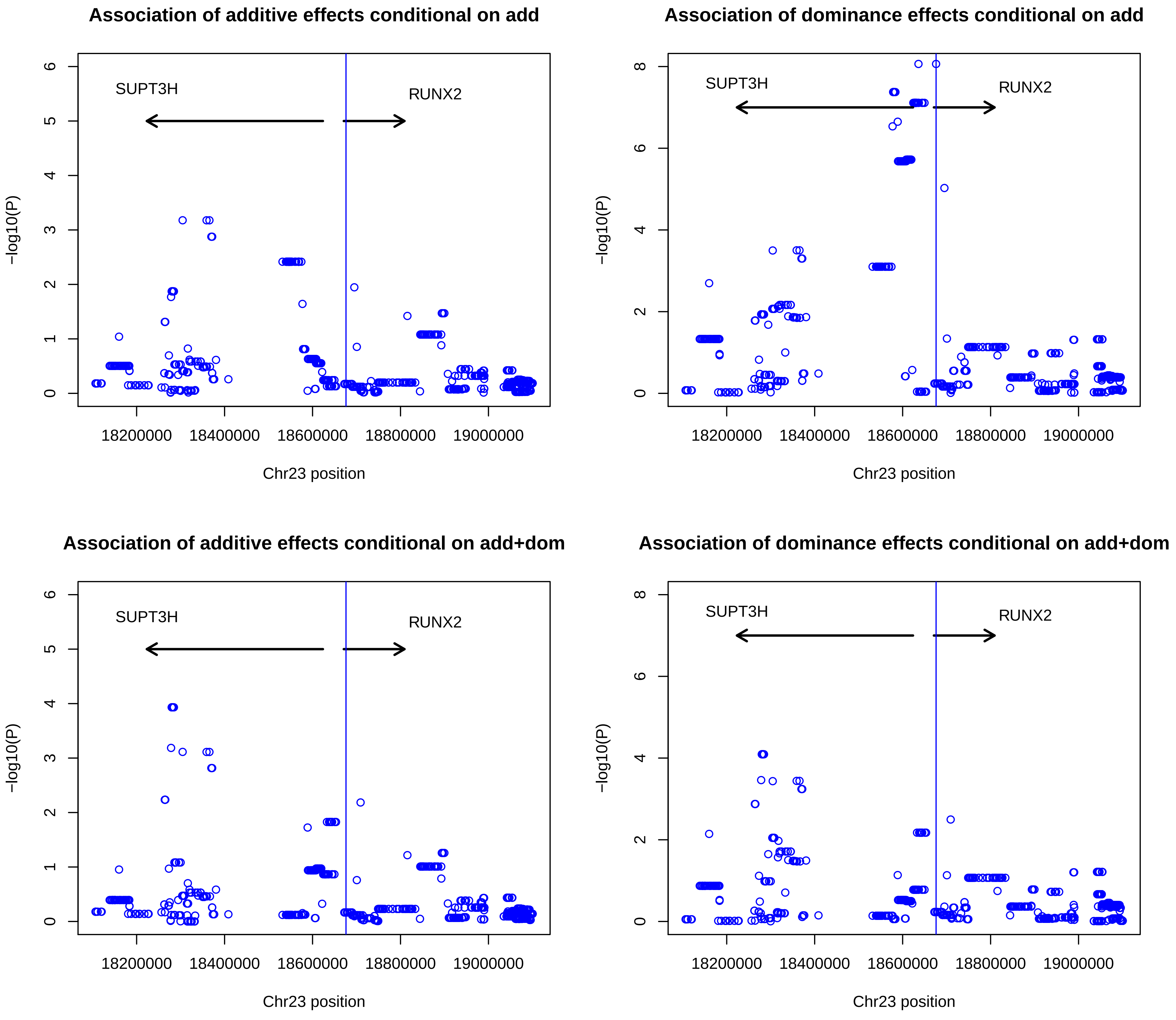

Association of additive effects conditional on add
Association of dominance effects conditional on add
Association of additive effects conditional on add+dom
Association of dominance effects conditional on add+dom
SUPT3H
RUNX2
−log10(P)
Chr23 position
18200000
18400000
18600000
18800000
19000000
0
1
2
3
4
5
6
8
